# *Lactobacillus rhamnosus* Lcr35 as an effective treatment for preventing *Candida albicans* infection in the invertebrate model *Caenorhabditis elegans*: First mechanistic insights

**DOI:** 10.1371/journal.pone.0216184

**Published:** 2019-11-06

**Authors:** Cyril Poupet, Taous Saraoui, Philippe Veisseire, Muriel Bonnet, Caroline Dausset, Marylise Gachinat, Olivier Camarès, Christophe Chassard, Adrien Nivoliez, Stéphanie Bornes

**Affiliations:** 1 Université Clermont Auvergne, INRA, VetAgro Sup, Aurillac, France; 2 Biose Industrie, Aurillac, France; Leibniz Institute for Natural Products Research and Infection Biology- Hans Knoell Institute, GERMANY

## Abstract

The increased recurrence of *Candida albicans* infections is associated with greater resistance to antifungal drugs. This involves the establishment of alternative therapeutic protocols, such as probiotic microorganisms whose antifungal potential has already been demonstrated using preclinical models (cell cultures, laboratory animals). Understanding the mechanisms of action of probiotic microorganisms has become a strategic need for the development of new therapeutics for humans. In this study, we investigated the prophylactic anti-*C*. *albicans* properties of *Lactobacillus rhamnosus* Lcr35^®^ using the *in vitro* Caco-2 cell model and the *in vivo Caenorhabditis elegans* model. In Caco-2 cells, we showed that the strain Lcr35^®^ significantly inhibited the growth (~2 log CFU.mL^-1^) and adhesion (150 to 6,300 times less) of the pathogen. Moreover, in addition to having a pro-longevity activity in the nematode (+42.9%, p = 3.56.10^−6^), Lcr35^®^ protects the animal from the fungal infection (+267% of survival, p < 2.10^−16^) even if the yeast is still detectable in its intestine. At the mechanistic level, we noticed the repression of genes of the p38 MAPK signalling pathway and genes involved in the antifungal response induced by Lcr35^®^, suggesting that the pathogen no longer appears to be detected by the worm immune system. However, the DAF-16/FOXO transcription factor, implicated in the longevity and antipathogenic response of *C*. *elegans*, is activated by Lcr35^®^. These results suggest that the probiotic strain acts by stimulating its host via DAF-16 but also by suppressing the virulence of the pathogen.

## 1 Introduction

*Candida albicans* is a commensal yeast found in the gastrointestinal and urogenital tracts [1,2] and is responsible for various diseases ranging from superficial infections affecting the skin to life-threatening systemic pathologic states i.e., candidemia [3]. Its pathogenicity is based on several factors, such as the formation of biofilms, thigmotropism, adhesion and invasion of host cells, secretion of hydrolytic enzymes [3] and the transition from yeast to hyphal filaments, which facilitates its spread [4,5].

There is an increase in the number of fungal infections, mainly due to the increaseraise in resistance to drugs [6,7] and to the limited number of available antifungals, some of which are toxic [8]. In addition, it is very common that antifungal treatments destabilize, more or less severely, the host commensal microbiota, leading to dysbiosis [9] which is favourable to the establishment of another pathogen or recurrence. In addition, because of the presence of similarities between yeasts and human cells (i.e., eukaryotic cells), the development of novel molecules combining antifungal activity and host safety is particularly complicated [8]. These different elements demonstrate the need to develop new therapeutic strategies. These aimed at effectively treating a fungal infection while limiting the health risks for the host; in particular, by preserving the integrity of its microbiota. The use of probiotics to cure candidiasis or fungal-infection-related dysbiosis is part of these novel strategies [10–12]. The World Health Organization (WHO) and the Food and Agriculture Organization of the United Nations (FAO) define probiotics as “live microorganisms, which, when administered in adequate amounts, confer a health benefit on the host” [13]. Under this appellation of probiotics, a wide variety of microbial species, are found within both prokaryotes and eukaryotes (yeasts, such as *Saccharomyces*), although these are mainly lactic bacteria, such as the genera *Lactobacillus* and *Bifidobacterium* [14]. Currently, a new name is increasingly used to replace the term probiotic: live biotherapeutic products (LBP). These LBP are biological products containing live biotherapeutic microorganisms (LBM) used to prevent, treat or cure a disease or condition of human beings, excluding vaccines [15].

In this issue, we focused on *L*. *rhamnosus* Lcr35^®^, which is a well-known probiotic strain whose *in vitro* and *in vivo* characteristics are widely documented [16–23]. It is a gram-positive bacterium commercialized by biose^®^ as a pharmaceutical product for more than 60 years for preventive and curative gastrointestinal and gynaecological indications. Nivoliez *et al*. demonstrated the probiotic properties of the native strain such as resistance to gastric acidity and bile stress and lactic acid production. Under its commercial formulations, the Lcr35^®^ strain has the ability to adhere to intestinal (Caco-2, HT29-MTX) and vaginal (CRL -2616) epithelial cells, but the inhibition of pathogen adhesion to intestinal cells by Lcr35^®^ has not been investigated by the authors. This study has also shown that Lcr35^®^ leads to a strong inhibition of vaginal (*C*. *albicans*, *Gardnerella vaginalis*) and intestinal (enterotoxigenic and enteropathogenic *Escherichia coli* (ETEC, EPEC), *Shigella flexneri*) pathogens [24]. These probiotic and antimicrobial effects have been observed during clinical trials, but we know little about the molecular mechanisms underlying these properties. Randomized trials conducted in infants and children have shown that preventive intake of probiotics has a positive impact on the development of infectious or inflammatory bowel diseases by reducing their symptoms and maintaining the balance of the microbiota [25]. *In vitro* and *in vivo* studies using preventive approaches have revealed certain mechanisms of action of probiotics [26].

Up to now, most probiotics used in both food and health applications are selected and characterized on the basis of their properties obtained with *in vitro* models [27] before being tested on complex *in vivo* models (murine models) and in human clinical trials. The *in vitro* studies are used mainly for ethical and cost issues [28] but also allow experimentations under defined and controlled conditions. As a result, some strains meeting the criteria for *in vitro* selection no longer respond *in vivo* and vice versa [29]. This fact reinforces the idea that *in vitro* and *in vivo* tests are complementary and necessary for the most reliable characterization of probiotic properties.

Here, we propose to use both *in vitro* Caco-2 cell culture and the invertebrate host *C*. *elegans* as an *in vivo* model to investigate microorganism-microorganism-host interactions. Caco-2 cells are a well-characterized enterocyte-like cell line. They are a reliable *in vitro* system to study the adhesion capacity of lactobacilli as well as their probiotic effects, such as protection against intestinal injury induced by pathogens [30,31]. Nevertheless, the use of *in vivo* models, which are closer to the complex environment of the human body, is inevitable in the case of a mechanistic study. Indeed, while rudimentary models such as *C*. *elegans* or *Drosophila* exhibit obvious benefits for (large) screening purposes, they are also not devoid of relevance in deciphering more universal signalling pathways, even related to mammalian innate immunity [32]. With its many genetic and protein homologies with human beings [33], *C*. *elegans* has become the ideal laboratory tool for physiological as well as mechanistic studies. This roundworm has already been used to study the pathogenicity mechanisms of *C*. *albicans*. The work of Pukkila-Worley has demonstrated a rapid antifungal response in *C*. *elegans* with the overexpression of antimicrobials encoding genes such as *abf-2*, *fipr-22*, *fipr-23*, *cnc-7*, *thn-1* and chitinases (*cht-1* and T19H5.1) or detoxification enzymes (*oac-31*, *trx-3*). It has also been shown that *C*. *albicans* hyphal formation is a key virulence factor that modifies gene expression in the *C*. *elegans* killing assay [34]. Some of these genes are notably dependent on the highly conserved p38 MAPK signalling pathway [35]. Several recent studies have established that the transition from yeast morphology to hyphal form is largely dependent on environmental parameters. It is also controlled by *C*. *albicans* genetic factors, such as eIF2 kinase Gcn2 [36] or SPT20 [37], whose mutations induce a decrease in virulence of the pathogen and an enhanced survival of the host. However, few studies have been conducted with the nematode on the use of probiotic microorganisms for the treatment of *C*. *albicans* fungal infection [38].

In this context, the aim of this study was to evaluate the effect of the *Lactobacillus rhamnosus* Lcr35^®^ strain on the prevention of fungal infection due to *C*. *albicans* using the *in vitro* cellular model Caco-2 and the *in vivo* model *C*. *elegans*. To overcome the experimental limits of the *in vitro* model, we conducted a mechanistic study solely on the *C*. *elegans* model. The worm survival and gene expression in response to the pathogen and/or the probiotic were evaluated.

## 2 Material and methods

### 2.1 Microbial strains and growth conditions

The *E*. *coli* OP50 strain was provided by the *Caenorhabditis* Genetics Center (Minneapolis, MN, USA) and was grown on Luria Broth (LB, Miller’s Modification) (Conda, Madrid, Spain) at 37 °C overnight. The *L*. *rhamnosus* Lcr35^®^ strain was provided by biose^®^ (Aurillac, France) and was grown in de Man, Rogosa, Sharpe (MRS) broth (bioMérieux, Marcy l’Etoile, France) at 37 °C overnight. *C*. *albicans* ATCC 10231 was grown in yeast peptone glucose (YPG) broth pH 6.5 (per L: 10 g yeast extract, 10 g peptone, 20 g glucose) at 37 °C for 48 h. Microbial suspensions were spun down for 2 min at 1,500 rpm (Rotofix 32A, Hettich Zentrifugen, Tuttlingen, Germany) and washed with M9 buffer (per L: 3 g KH_2_PO_4_, 6 g Na_2_HPO_4_, 5 g NaCl, 1 mL 1 M MgSO_4_) to obtain a final concentration of 100 mg.mL^-1^.

### 2.2 Influence of Lcr35^®^ on *C*. *albicans* growth and on *C*. *albicans* biofilm formation on Caco-2 cell monolayers

Growth inhibition of *C*. *albicans* by the probiotic strain Lcr35^®^ was examined using the human colorectal adenocarcinoma cell line Caco-2 [39]. Caco-2 cells were grown in Dulbecco’s modified Eagle’s minimal essential medium (DMEM, Life Technologie, Villebon-sur-Yvette, France) supplemented with 20% inactivated foetal calf serum (Life Technologie) at 37 °C with 5% CO_2_ in air atmosphere. For the assays, the cells were seeded at a concentration of 3.5x10^5^ cells.well^-1^ in 24-well plates (Dutscher, Brumath, France) and placed in growth conditions for 24 h. Microbial strains were grown according to Nivoliez *et al*. [24]. After growth, cell culture medium was removed and replaced by 1 mL of DMEM and 250 μL of Lcr35^®^ culture (10^8^ CFU.mL^-1^) in each well and incubated for 24 h. Two hundred and fifty microliters of *C*. *albicans* culture at different concentrations (10^2^, 10^3^, 10^4^, 10^5^, 10^6^ and 10^7^ CFU.mL^-1^) were added to each well. After incubation for 24 and 48 h, the inhibition of *C*. *albicans* by Lcr35^®^ was evaluated. One hundred microliters of suspension were taken from each of the wells, and the number of viable bacteria and/or yeasts was determined by plating serial dilutions of the suspensions onto MRS or Sabouraud agar plates. For the measurement of *C*. *albicans* biofilm formation, after incubation for 48 h, the wells were washed twice with 0.5 mL of PBS and cells were harvested with 1 mL of trypsin at 37 °C. For the inhibition assay, the number of viable bacteria and/or yeasts was determined by plating serial dilutions of the suspensions onto MRS or Sabouraud agar plates. The plates were incubated at 37 °C for 72 h (MRS) or 48 h (Sabouraud). Each assay was performed three times independently and contained two technical replicates.

### 2.3 *C*. *elegans* maintenance

*C*. *elegans* N2 (wild-type) and TJ356 (*daf-16p*::*daf-16a/b*::*GFP* + *rol-6(su1006)*) strains were acquired from the *Caenorhabditis* Genetics Center. The nematodes were grown and maintained at 20 °C on nematode growth medium (NGM) (per L: 3 g NaCl; 2.5 g peptone; 17 g agar; 5 mg cholesterol; 1 mM CaCl_2_; 1 mM MgSO_4_, 25 mL 1 M potassium phosphate buffer at pH 6) plates supplemented with yeast extract (4 g.L^-1^) (NGMY) and seeded with *E*. *coli* OP50 [40]. For all experiments, wild-type *C*. *elegans* N2 were used except for the study of the localization of DAF-16 (TJ356 strain).

### 2.4 *C*. *elegans* synchronization

To avoid variations in results due to age differences, a worm synchronous population was required. Gravid worms were washed off using M9 buffer and spun down for 2 min at 1,500 rpm. Five millilitres of worm bleach (2.5 mL of M9 buffer, 1.5 mL of bleach, 1 mL of 5 M sodium hydroxide) was added to the pellet and vigorously shaken until adult worm body disruption. The action of worm bleach was stopped by adding 20 mL of M9 buffer. The egg suspension was then spun down for 2 min at 1,500 rpm and washed twice with 20 mL of M9 buffer. Eggs were allowed to hatch under slow agitation at 25 °C for 24 h in approximately 20 mL of M9 buffer. L1 larvae were then transferred onto NGMY plates seeded with *E*. *coli* OP50 until they reached the L4/young adult stage.

### 2.5 *C*. *elegans* bodyb size measurement

Individual adult worms were imaged using an Evos FL microscope (Invitrogen, Eugene, USA, 10X magnification). After reaching the L4 stage, they were transferred onto NGMY plates previously seeded with the probiotic strain Lcr35^®^, and their sizes were measured daily for three days. The length of the worm body was determined using ImageJ software as described by Mörck and Pilon (41) and compared to *E*. *coli* OP50-fed worms. At least 10 nematodes per experiment were imaged in at least three independent experiments.

### 2.6 *C*. *elegans* lifespan assay

Synchronous L4 worms were transferred to NGMY with 0.12 mM 5-fluorodeoxyuridine FUdR (Sigma, Saint-Louis, USA) to avoid egg hatching and seeded with 100 μL of microbes at 100 mg.mL^-1^ microbial strain (~50 worms per plate) as previously stated. The plates were kept at 20 °C, and live worms were scored each day until the death of all animals. An animal was scored as dead when it did not respond to a gentle mechanical stimulation. This assay was performed as three independent experiments with three plates per condition.

### 2.7 Effects of *L*. *rhamnosus* Lcr35^®^ on candidiasis in *C*. *elegans*

Sequential feeding with Lcr35^®^ and *C*. *albicans* were induced in *C*. *elegans* in all experiments (preventive assays). As control groups, monotypic contamination was induced in *C*. *elegans* by inoculation with only *C*. *albicans*, Lcr35^®^ or *E*. *coli* OP50.

#### 2.7.1 Preparation of plates containing probiotic bacteria or pathogenic yeasts

One hundred microliters of Lcr35^®^ or *E*. *coli* OP50 suspension (100 mg.mL^-1^) was spread on NGMY + 0.12 mM FUdR plates and incubated at 37 °C overnight. Concerning *C*. *albicans* strains, 100 μL of suspension was spread on Brain Heart Infusion BHI (Biokar Diagnostics, Beauvais, France) + 0.12 mM FUdR plates and incubated at 37 °C overnight.

#### 2.7.2 Survival assay: Preventive treatment

The survival assay was performed according to the work of de Barros [38], with some modifications. During a preventive treatment, young adult worms were placed on plates containing Lcr35^®^ at 20 °C for different times (2, 4, 6 and 24 h). Next, the worms were washed with M9 buffer to remove bacteria prior to being placed on *C*. *albicans* plates for 2 h at 20 °C. Infected nematodes were washed off plates using M9 buffer prior to being transferred to a 6-well microtiter plate (approximately 50 worms per well) containing 2 mL of BHI/M9 (20%/80%) + 0.12 mM FUdR liquid assay medium per well and incubated at 20 °C. For the control groups (i.e., *E*. *coli* OP50 + *C*. *albicans*, *E*. *coli* OP50 only, Lcr35^®^ only and *C*. *albicans* only), worms were treated in the same way. Nematodes were observed daily and were considered dead when they did not respond to a gentle mechanical stimulation. This assay was performed as three independent experiments containing three wells per condition.

### 2.8 Colonization of *C*. *elegans* intestine by *C*. *albicans*

To study the colonization of the worm gut by the pathogen *C*. *albicans*, fluorescent staining of the yeast was performed. The yeast was stained with rhodamine 123 (Yeast Mitochondrial Stain Sampler Kit, Invitrogen) according to the manufacturer’s instructions. A fresh culture of *C*. *albicans* was performed in YPG broth as described before, 1.6 μL of rhodamine 123 at 25 mM was added to 1 mL of *C*. *albicans* suspension and incubated at room temperature in the dark for 15 min. The unbound dye was removed by centrifugation (14,000 rpm for 5min at 4 °C) (Beckman J2-MC Centrifuge, Beckman Coulter, Brea, USA) and washed with 1 mL of M9 buffer. Subsequently, the nematodes were fed with *E*. *coli* OP50 or Lcr35^®^ on NGMY plates for 4 h and then with labelled *C*. *albicans* on BHI plates for 72 h. The nematodes were then visualized using a fluorescence microscope at 100X magnification (Evos FL, Invitrogen).

### 2.9 RNA isolation and RT- quantitative PCR

Approximately 10,000 worms were harvested from NGMY plates with M9 buffer. Total RNA was extracted by adding 500 μL of TRIzol reagent (Ambion by Life Technologies, Carlsbad, USA). Worms were disrupted using a Precellys (Bertin Instruments, Montigny-le-Bretonneux, France) and glass beads (PowerBead Tubes Glass 0.1 mm, Mo Bio Laboratories, USA). Beads were removed by centrifugation at 14,000 rpm for 1 min (Eppendorf^®^ 5415D, Hamburg, Germany), and 100 μL of chloroform was added to the supernatant. Tubes were vortexed for 30 seconds and incubated at room temperature for 3 minmin. The phenolic phase was removed by centrifugation at 12,000 rpm for 15 min at 4 °C. The aqueous phase was treated with chloroform as previously described. RNA was precipitated by adding 250 μL of isopropanol for 4 min at room temperature and spun down at 12,000 rpm for 10 min (4 °C). The supernatant was discarded, and the pellet was washed with 1,000 μL of 70% ethanol. The supernatant was discarded after centrifugation at 14,000 rpm for 5 min (4 °C), and the pellet was dissolved in 20 μL of RNase-free water. RNA was reverse-transcribed using a High-Capacity cDNA Archive kit (Applied Biosystems, Foster City, USA) according to the manufacturer’s instructions. For real-time qPCR assay, each tube contained 2.5 μL of cDNA, 6.25 μL of Rotor-Gene SYBR Green Mix (Qiagen GmbH, Hilden, Germany), 1.25 μL of 10 μM primers (reported in Table 1) (Eurogentec, Seraing, Belgium) and 1.25 μL of water. All samples were run in triplicate. Rotor-Gene Q Series Software (Qiagen GmbH) was used for the analysis. In our study, two reference genes, *cdc-42* and Y45F10D.4, were used in all the experimental groups. The quantification of gene-of-interest expression (E_GOI_) was performed according to the following formula [41] taking into account the efficiency of the PCR for each primer pair and normalizing the expression of the gene of interest by two reference genes (*cdc-42* and Y45F10D.4):

$$E_{GOI}=\frac{{\left(GOI\;efficiency\right)}^{\Delta Ct_{GOI}}}{\sqrt{{\left(cdc-42efficiency\right)}^{\Delta Ct_{cdc-42}}\times {\left(Y45F10D.4\;efficiency\right)}^{\Delta Ct_{Y45F10D.5}}}}$$

**Table 1:** *C*. *elegans* gene primers for qPCR analysis. GOI: Gene of interest.

Gene name	Gene type	Forward Primer (5’– 3’)	Reverse Primer (5’– 3’)	Reference
*cdc-42*	housekeeping	ATCCACAGACCGACGTGTTT	GTCTTTGAGCAATGATGCGA	[42]
Y45F10D.4	housekeeping	CGAGAACCCGCGAAATGTCGGA	CGGTTGCCAGGGAAGATGAGGC	[43]
*daf-2*	GOI	AAAAGATTTGGCTGGTCAGAGA	TTTCAGTACAAATGAGATTGTCAGC	[44]
*daf-16*	GOI	TTCAATGCAAGGAGCATTTG	AGCTGGAGAAACACGAGACG	[44]
*sek-1*	GOI	GCCGATGGAAAGTGGTTTTA	TAAACGGCATCGCCAATAAT	[44]
*pmk-1*	GOI	CCGACTCCACGAGAAGGATA	AGCGAGTACATTCAGCAGCA	[44]
*abf-2*	GOI	TCGTCCGTTCCCTTTTCCTT	CCTCTCTTAATAAGAGCACC	This study
*fipr-22/fipr-23*	GOI	CCCAATCCAGTATGAAGTTG	ATTTCAGTCTTCACACCGGA	This study
*cnc-4*	GOI	ATGCTTCGCTACATTCTCGT	TTACTTTCCAATGAGCATTC	This study

The worms fed with *E*. *coli* OP50 were used as control conditions for the gene expression calculation.

### 2.10 Statistical analysis

Data are expressed as the mean ± standard deviation.

The *C*. *elegans* survival assay was examined using the Kaplan-Meier method, and differences were determined using the log-rank test with R software version 3.5.0 [45], and the *survival* [46] and *survminer* [47] packages. For *C*. *albicans* growth inhibition and biofilm formation and *C*. *elegans* growth and gene expression of the genes analysed, differences between conditions were determined by a two-way ANOVA followed by a Fisher’s Least Significant Difference (LSD) post hoc test using GraphPad Prism version 7.0a for Mac OS X (GraphPad Software, La Jolla, California, USA). A *p*-value ≤ 0.05 was considered significant.

### 2.11 DAF-16 nuclear localization

DAF-16 nuclear localization was followed as described elsewhere [48] using a transgenic TJ-356 worm strain constitutively expressing the DAF-16 transcription factor combined with GFP (DAF-16::GFP). Once adults, worms were exposed to a single strain: *E*. *coli* OP50, Lcr35^®^ or *C*. *albicans* for 2, 4, 6, 24 and 76 h at 20 °C. A preventive approach was also conducted: worms were placed in the presence of *E*. *coli* OP50 or Lcr35^®^ for 4 h and then *C*. *albicans* for 2 hh. The nematodes were subsequently imaged 2, 4, 6 and 24 h after infection. The translocation of DAF-16::GFP was scored by assaying the presence of GFP accumulation in the *C*. *elegans* cell nuclei using a fluorescence microscope at 40X magnification (Evos FL, Invitrogen).

## 3 Results

### 3.1 Anti-*C*. *albicans* effects of Lcr35^®^ on Caco-2 cell monolayer

#### 3.1.1 Growth inhibition of *CC*. *albicans*

In the presence of Caco-2 cells, regardless of the concentration of the *C*. *albicans* inoculum, the yeast grew to similar concentrations that ranged from 7.48 ± 0.39 to 7.83 ± 0.34 log CFU.mL^-1^ after 48 h of incubation. When prophylactic treatment was used, i.e., when the Caco-2 cells were pre-incubated with the probiotic Lcr35^®^, we observed an inhibition of *C*. *albicans* growth. Indeed, the bacterium induced a significant inhibition of the yeast growth of 2 log CFU.mL^-1^, which then reached a concentration ranging from 5.40 ± 0.07 to 6.05 ± 0.25 log CFU.mL^-1^. Two different inhibition profiles were observed after 48 h. On the one hand, when the inoculum was highly concentrated (7 log CFU.mL^-1^), we observed a decrease in the yeast population, which is a sign of cell death. On the other hand, when the inoculum was less concentrated (2 to 4 log CFU.mL^-1^), we noticed that the yeast was able to grow, although its growth seemed to stop between 5.32 ± 0.36 and 5.51 ± 0.14 log CFU.mL^-1^ (Table 2).

**Table 2:** Evolution of the concentration of *C*. *albicans* in the presence or absence of Lcr35^®^ on Caco-2 cell monolayers.

		Length of incubation (hh)		
Concentration of *C*. *albicans* inocula (CFU.mL^-1^)	With or without Lcr35^®^	0	24	48
**10**^**7**^	**with**	7.25 ± 0.51	6.39 ± 0.73	6.05 ± 0.25 ****
	**without**	6.77 ± 0.10	7.29 ± 0.23	7.78 ± 0.41
**10**^**6**^	**with**	5.85 ± 0.25	5.47 ± 0.12 *	5.73 ± 0.09 ***
	**without**	5.76 ± 0.18	7.42 ± 0.27	7.69 ± 0.20
**10**^**5**^	**with**	4.77 ± 0.41	5.01 ± 0.12 **	5.49 ± 0.04 ****
	**without**	4.60 ± 0.28	7.60 ± 0.69	7.83 ± 0.34
**10**^**4**^	**with**	3.69 ± 0.21	4.92 ± 0.54	5.51 ± 0.14 *
	**without**	3.72 ± 0.13	7.09 ± 0.59	7.48 ± 0.39
**10**^**3**^	**with**	2.56 ± 0.34	3.59 ± 0.25	5.51 ± 0.16 ****
	**without**	2.30 ± 0.17	6.60 ± 0.28	7.93 ± 0.45
**10**^**2**^	**with**	1.34 ± 0.31	3.18 ± 0.76	5.32 ± 0.36 ***
	**without**	1.34 ± 0.38	6.18 ± 1.01	7.80 ± 0.27

The results are expressed as log_10_ CFU.mL^-1^ of yeast alone (controls) or co-incubated with Lcr35^®^ (mean ± standard deviation). A comparison between the conditions with and without Lcr35^®^ was performed using a two-way ANOVA followed by a Fisher’s LSD post hoc test

(p < 0.05: *; p < 0.01: **; p < 0.001: ***; p < 0.0001: ****)

#### 3.1.2 Inhibition of *C*. *albicans* biofilm formation

**Fig 1:**
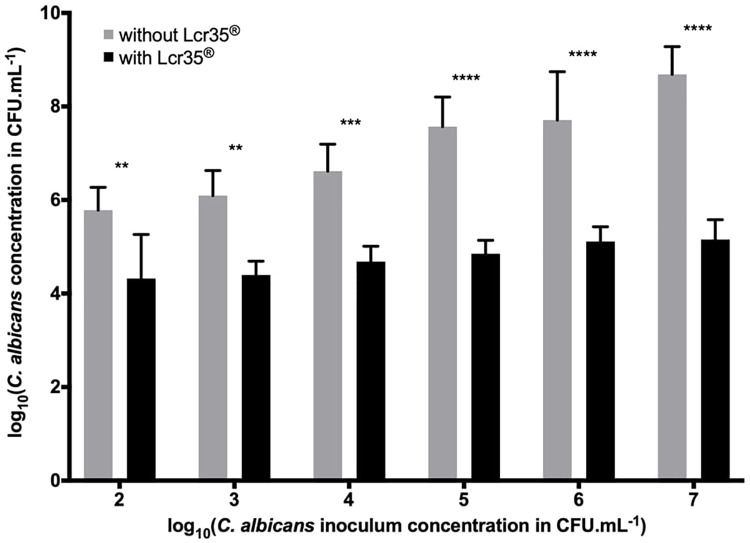
Determination of the *C*. *albicans* concentration in the biofilm in the presence or absence of Lcr35^®^ (10^8^ CFU.mL^-1^) on the Caco-2 cell monolayer (mean ± standard deviation). Different concentrations of yeast were tested, and the amount present in the biofilm was evaluated after 48 h of incubation. Comparison between conditions with and without Lcr35^®^ was performed using a two-way ANOVA followed by a Fisher’s LSD post hoc test (p < 0.05: *; p < 0.01: **; p < 0.001: ***; p < 0.0001: ****).

After 48h of incubation, the *C*. *albicans* biofilm contained between 5.78 log CFU.mL^-1^ (inoculum at 10^2^ CFU.mL^-1^) and 8.69 log CFU.mL^-1^ of yeast (inoculum at 10^7^ CFU.mL^-1^). However, since the cells were pre-exposed to Lcr35^®^ and for the same *C*. *albicans* inocula, we observed a significant decrease in the amount of yeast in the biofilm: 4.32 to 5.16 log CFU.mL^-1^, which corresponded to an inhibition ranging from 1.46 to 3.53 log. The strongest inhibition was observed in the case where the inoculum of *C*. *albicans* was the most concentrated (Fig 1).

### 3.2 Effects of Lcr35^®^ on *C*. *elegans* physiology

#### 3.2.1 Lcr35^®^ extends the *C*. *elegans* lifespan

**Fig 2:**
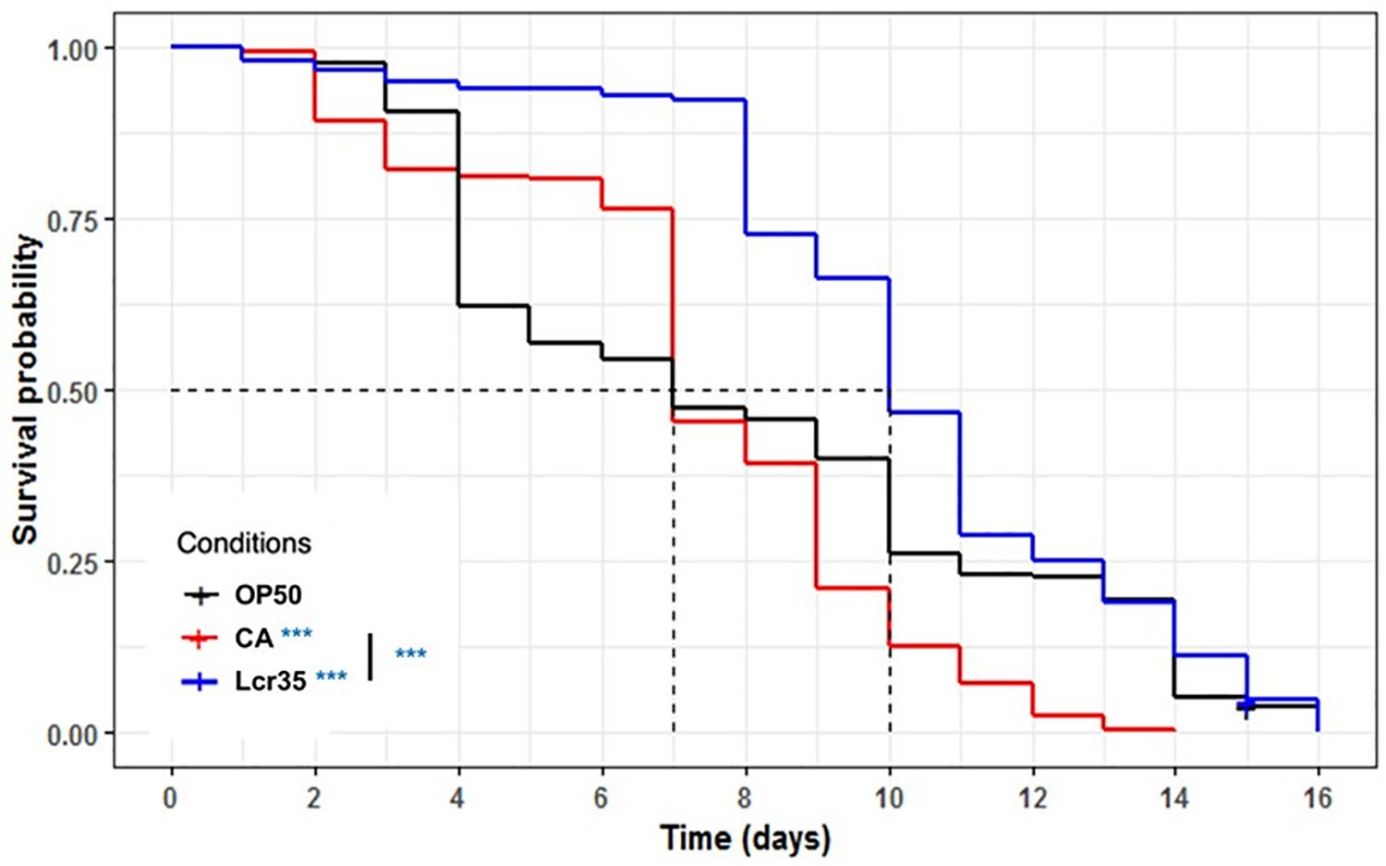
Influence of *Lactobacillus rhamnosus* Lcr35^®^ and *C*. *albicans* on the lifespan of the *C*. *elegans* wild-type N2 strain. Worms were fed *E*. *coli* OP50 (n = 285), *C*. *albicans* ATCC 10231 (n = 242), and Lcr35^®^ (n = 278). The mean lifespan, where half of the population was dead, is represented on the abscissa. The asterisks indicate the *p-values* (log-rank test) with *E*. *coli* OP50 as a control (p < 0.05: *; p < 0.01: **; p < 0.001: ***).

We investigated the effects on *C*. *elegans* lifespan induced by either the pathogenic yeast *C*. *albicans* or the probiotic Lcr35^®^. Feeding adult nematodes with the probiotic strain resulted in a significant increase in the mean lifespan compared to *E*. *coli* OP50-fed worms (p = 3.56.10^−6^) evolving from 7 to 10 days (+ 42.9%), whereas *C*. *albicans* had no impact on the mean lifespan of *C*. *elegans*. On the other hand, when *C*. *albicans* was used as a feeding source, worms displayed a significantly reduced longevity (p = 1.27.10^−5^), which dropped from 16 to 14 days (-12.5%). Lcr35^®^ did not increase the worm longevity compared to *E*. *coli* OP50 (Fig 2). These results showed that the probiotic strain ameliorated the mean lifespan without increasing the life expectancy of the worm.

#### 3.2.2 Lcr35^®^ does not modify *C*. *elegans* growth

**Fig 3:**
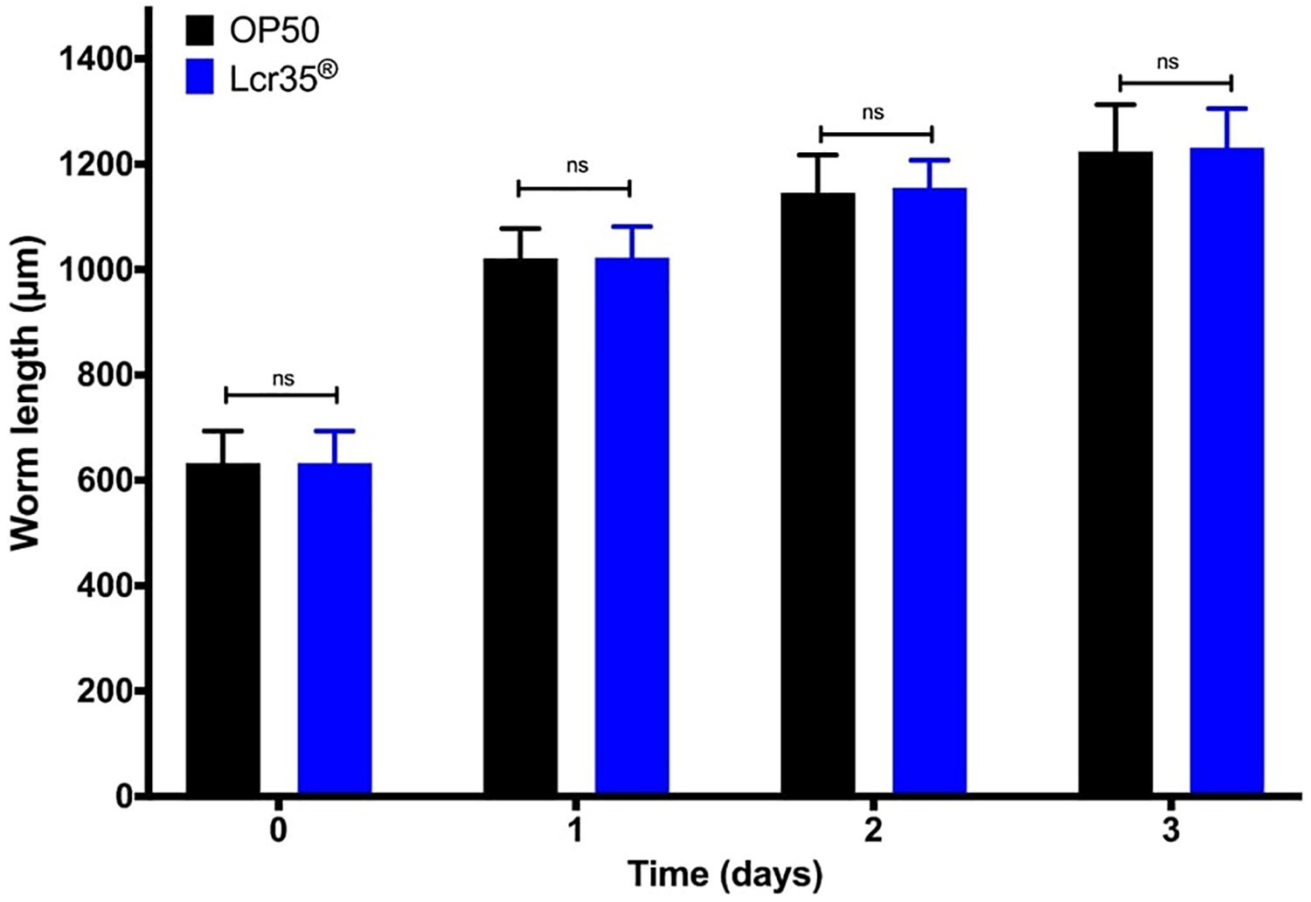
Growth of *C*. *elegans* (adult) on *E*. *coli* OP50 and on Lcr35^®^. All results are represented as means +/- standard deviations (ns: statistically not significant).

The body size of Lcr35^®^ fed nematodes was compared to that of *E*. *coli* OP50-fed worms. Feeding worms with the probiotic strain did not significantly change the growth rate or body size, as they all reached their maximal length after three days (Fig 3).

### 3.3 Effect of Lcr35^®^ preventive treatment on candidiasis

#### 3.3.1 Effect of Lcr35^®^ on *C*. *elegans* survival after *C*. *albicans* exposure

**Fig 4:**
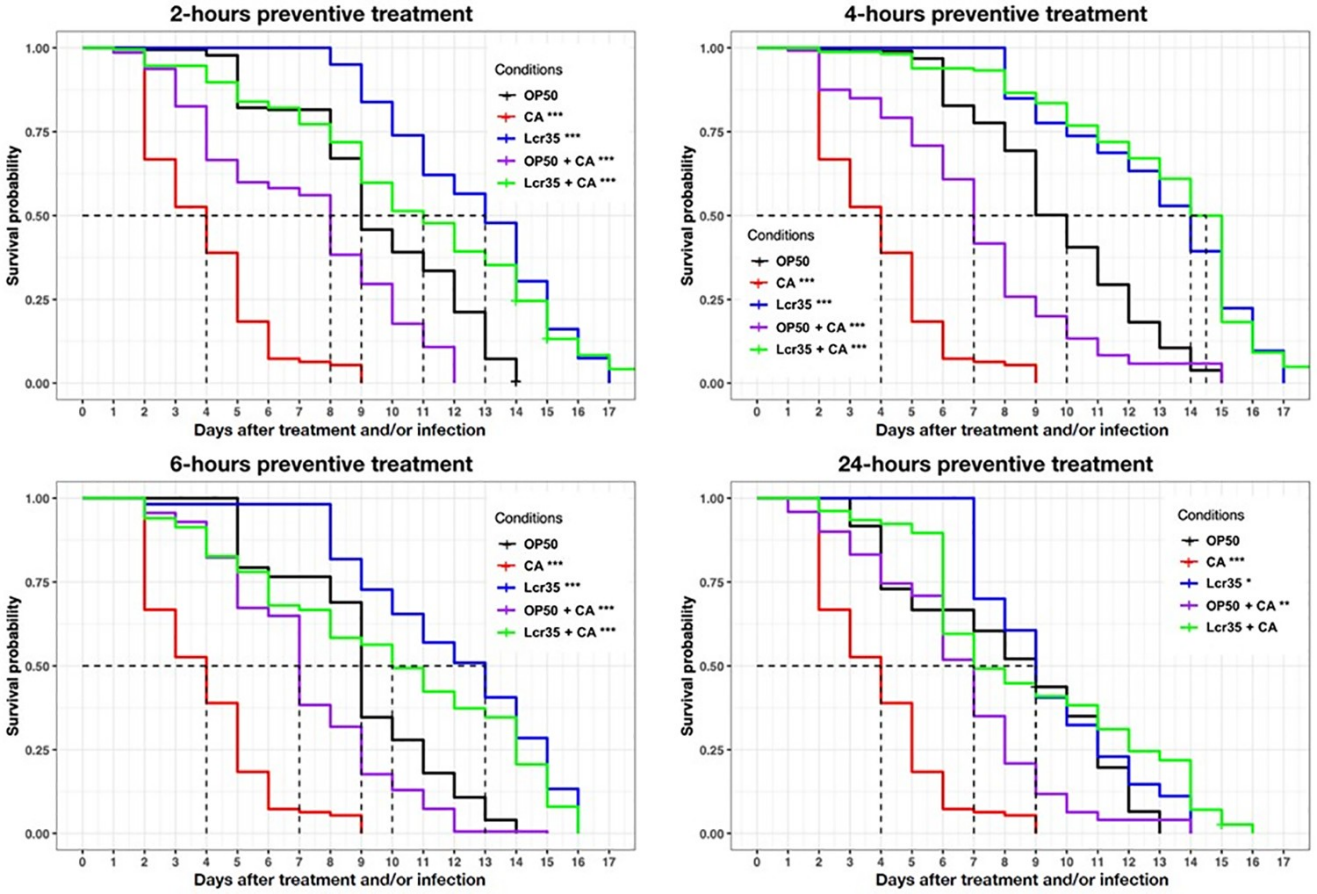
Preventive effects of Lcr35^®^ against *C*. *albicans* ATCC 10231. Mean survival, where half of the population was dead, is represented on the abscissa. The asterisks indicate the *p-values* (log-rank test) against *E*. *coli* OP50 (p < 0.05: *; p < 0.01: **; p < 0.001: ***). Infection duration: 2 hour;**2-hour preventive treatment** (*E*. *coli* OP50 (OP50, n = 126); *C*. *albicans* ATCC 10231 (CA, n = 424); Lcr35^®^ (Lcr35, n = 93); *E*. *coli* OP50 + *C*. *albicans* (OP50 + CA, n = 287); Lcr35^®^ + *C*. *albicans* (Lcr35 + CA, n = 224));**4-hour preventive treatment** (*E*. *coli* OP50 (OP50, n = 313); *C*. *albicans* ATCC 10231 (CA, n = 424); Lcr35^®^ (Lcr35, n = 259); *E*. *coli* OP50 + *C*. *albicans* (OP50 + CA, n = 120); Lcr35^®^ + *C*. *albicans* (Lcr35 + CA, n = 164)); **6-hour preventive treatment** (*E*. *coli* OP50 (OP50, n = 222); *C*. *albicans* ATCC 10231 (CA, n = 424); Lcr35^®^ (Lcr35, n = 165); *E*. *coli* OP50 + *C*. *albicans* (OP50 + CA, n = 339); Lcr35^®^ + *C*. *albicans* (Lcr35 + CA, n = 300)); **24-hour preventive treatment** (*E*.**treatment** *coli* OP50 (OP50, n = 248); *C*. *albicans* ATCC 10231 (CA, n = 424); Lcr35^®^ (n = 170); *E*. *coli* OP50 + *C*. *albicans* (OP50 + CA, n = 220); Lcr35^®^ + *C*. *albicans* (Lcr35 + CA, n = 183)).

When *C*. *elegans* was sequentially exposed to Lcr35^®^ for 2 h prior to being infected by *C*. *albicans*, the survival of the nematodes increased significantly as the mean lifespan increased from 3 to 11 days (267% increase in survival) compared with that observed with *C*. *albicans* infection alone (p < 2.10^−16^). There was no significant difference in worm survival between those sequentially exposed to Lcr35^®^ and *C*. *albicans* and those exposed to Lcr35^®^ only (Fig 4) (p = 1). Similar results were obtained with the 4-hours treatment time. In that case, we observed that Lcr35^®^ completely protected *C*. *elegans* from infection since there was no significant difference with the Lcr35^®^ control condition without infection (p = 0.4).

For longer treatment times (6 and 24 h), we observed a significant decrease in the mean survival in the presence of Lcr35^®^ (condition 6 h: p = 0.04, condition 24 h: p <2.10^−16^) or Lcr35^®^ and *C*. *albicans* (condition 6 h: p = 9.10^−13^, condition 24 h: p < 2.10^−16^) compared to the treatment of 4 h. Taken together, the results showed that the 4 probiotic treatment was the most protective against infection.

#### 3.3.2 Influence of Lcr35^®^ on *C*. *albicans* colonization of the worm gut

**Fig 5:**
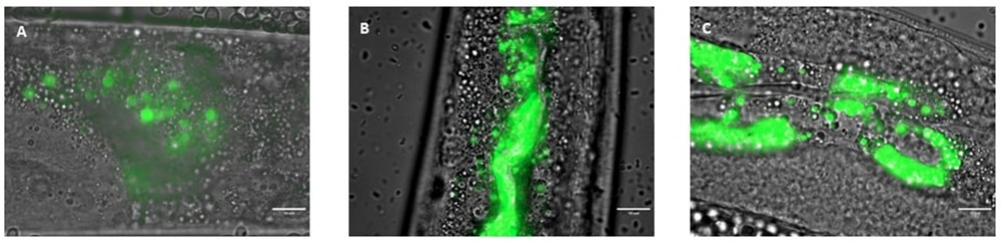
*C*. *albicans* colonization of the *C*. *elegans* gut after 72 h (A) and after a 4-hour prophylactic treatment with *E*. *coli* OP50 (B) or Lcr35^®^ (C). The green colour represents yeast labelled with rhodamine 123. Scale bar, 10 μm.

To determine whether the anti-*C*. *albicans* effects observed were due to the removal of the pathogen, colonization of the nematode intestine by *C*. *albicans* was observed by light microscopy. After three days of incubation in the presence of the pathogen, wild-type worms exhibited notable colonization of the entire digestive tract (Fig 5A). However, this strain of *C*. *albicans* was not able to form hyphae within the worm. We subsequently applied prophylactic treatment to the worms for 4 h before infecting them with yeast. We observed that after treatment with *E*. *coli* OP50 (Fig 5B) or the probiotic Lcr35^®^ (Fig 5C) followed by infection, the yeast *C*. *albicans* was still detected in the digestive tract of the host.

### 3.4 Mechanistic study

#### 3.4.1 Modulation of *C*. *elegans* gene expression induced by Lcr35^®^ and *C*. *albicans*

To elucidate the mechanisms involved in the action of Lcr35^®^ against *C*. *albicans*, we studied the expression of seven *C*. *elegans* genes (Table 3). We targeted three groups of genes: *daf-2* and *daf-16* (insulin signalling pathway), which are involved in host longevity and anti-pathogenicity; *sek-1* and *pmk-1* (p38 MAPK signalling pathway), which concern the immune response; and *abf-2*, *cnc-4* and *fipr-22/fipr-23*, which encode antimicrobial proteins. We noted that Lcr35^®^ tended to induce an overexpression of *daf-16* (p = 0.1635) and had no effect on *daf-2* (p = 0.2536), while *C*. *albicans* tended to induce an upregulation of both genes (p = 0.1155 and p = 0.2396, respectively). We did not observe any expression modulation of *daf-2* or *daf-16* using a preventive treatment with *E*. *coli* OP50 (p = 0.1258 and p = 0.1215, respectively) or with Lcr35^®^ (p = 0.1354 and p = 0.3021, respectively).

**Table 3:** Relative expression of the *C*. *elegans* genes of interest in the presence of Lcr35^®^ and *C*. *albicans* in pure or sequential cultures in comparison with the control condition *E*. *coli* OP50 (alone).

	Genes of interest						
	Insulin signalling pathway		p38 MAPK signalling pathway		Antimicrobials		
Conditions	*daf*-*2*	*daf-16*	*sek-1*	*pmk-1*	*abf-2*	*cnc-4*	*fipr-22* / *fipr-23*
**Lcr35**^**®**^	1.35	2.18	0.38 **	0.36 *	1.70	3.39	0.61
***C*. *albicans***	2.48	3.31	3.21 *	4.33	11.33	22.32	1.08
***E*. *coli* OP50 + *C*. *albicans***	1.82	0.53	0.37 *	3.40	4.69	0.16 **	0.78
**Lcr35**^**®**^ **+ *C*. *albicans***	0.69	1.74	0.31 **	1.15	1.61	0.41 *	0.42 *

Genes were considered differentially expressed when the p-value was lower than 0.05 (*) or 0.01 (**) according to Fisher’s LSD test and simultaneously when the expression change was at least 2 times or 0.5 times.

The expression of the *sek-1* and *pmk-1* immunity genes was significantly downregulated in the presence of Lcr35^®^ by 2.63-fold (p = 0.015) and 2.78-fold (p = 0.0149), respectively, while they were upregulated by *C*. *albicans* 3.21-fold (p = 0.0247) and 4.33-fold (0.1618), respectively. In the control condition, in the presence of *E*. *coli* OP50 and *C*. *albicans*, *sek-1* was repressed 2.70 times (0.37-fold with p = 0.0204), but *pmk-1* tended to be overexpressed. Preventive treatment with Lcr35^®^ had the same effect on *sek-1* (p = 0.0016) but induced no change in *pmk-1* expression (p = 0.8205). Finally, among the 3 antimicrobials encoding the genes tested, only the expression of *cnc-4* seemed to be modulated in the presence of Lcr35^®^, and *cnc-4* was overexpressed (p = 0.1753). *C albicans* also seemed to induce the overexpression of *abf-2* (p = 0.2213) and *cnc-4* (p = 0.3228), but interestingly, *fipr-22*/*fipr-23* (p = 0.8225) expression remained unchanged. Overexpression of *abf-2* (6.25-fold, p = 0.3158) and significant repression of *cnc-4* (p = 0.0088) were observed when *E*. *coli* OP50 was added before infection with *C*. *albicans*. Using a Lcr35^®^ preventive treatment, *cnc-4* and *fipr-22*/*fipr-23* were significantly repressed (p = 0.0396 and p = 0.0385, respectively).

#### 3.4.2 Influence of Lcr35^®^ and *C*. *albicans* on DAF-16 nuclear translocation

**Fig 6:**
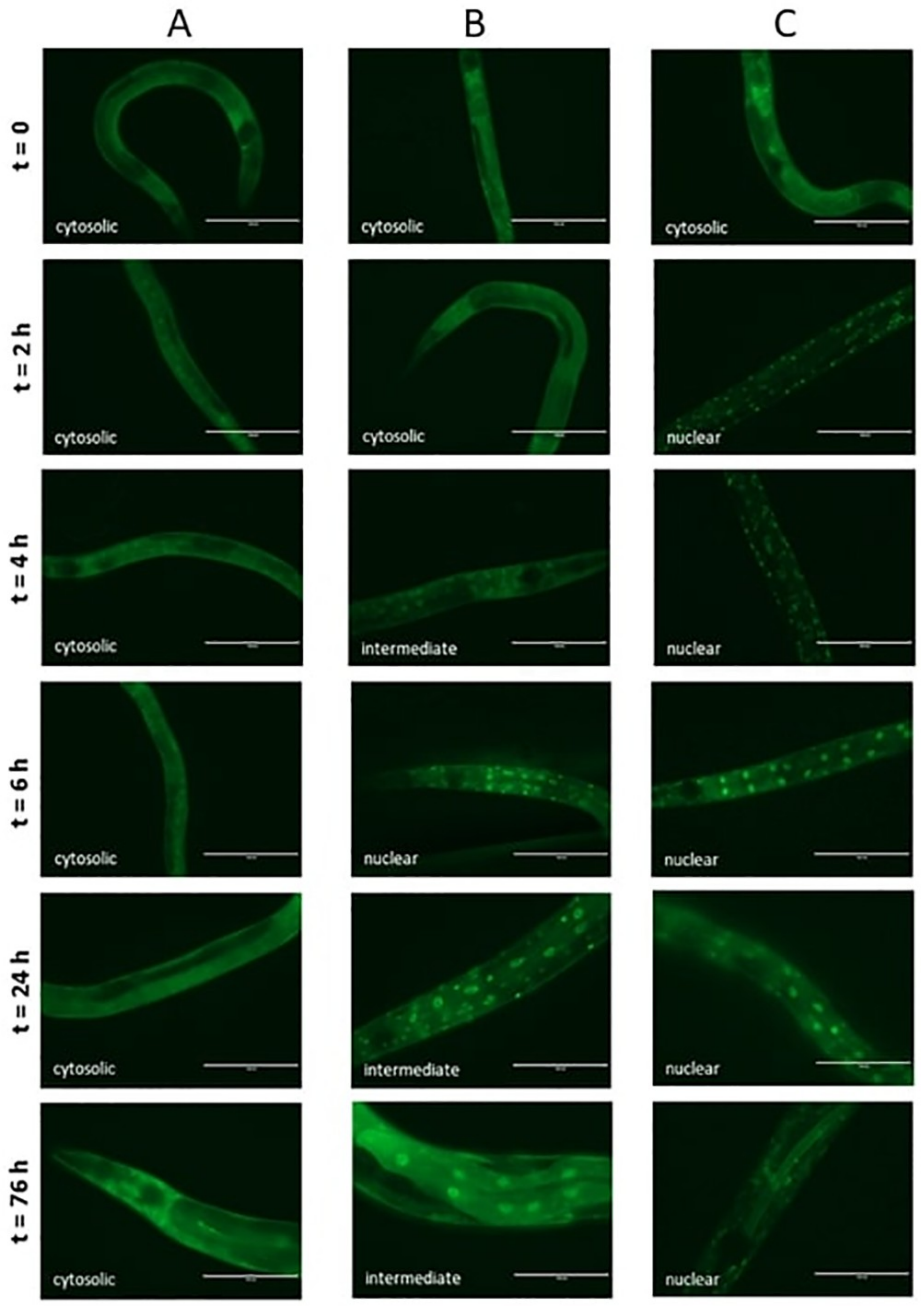
DAF-16 cellular localization in *C*. *elegans* transgenic strain TJ-356 (*daf-16p*::*daf-16a/b*::*GFP* + *rol-6(su1006)*) expressing DAF-16::GFP. Worms fed on *E*. *coli* OP50 (A), on Lcr35^®^ (B) and on *C*. *albicans* ATCC 10231 (C). Scale bar, 100 μm.

To further investigate the mechanisms involved in the anti-*C*. *albicans* effects of Lcr35^®^, we followed the nuclear translocation of the DAF-16/FOXO transcription factor using the DAF-16::GFP strain. Whatever the incubation time, the worms did not show any translocation of DAF-16 when fed with *E*. *coli* OP50 (Fig 6A). When Lcr35^®^ was used as food, we observed a nuclear translocation of the transcription factor, taking place gradually from 4 h of incubation with a maximum intensity in the nuclei after 6 hh. The distribution of DAF-16 was both cytoplasmic and nuclear (Fig 6B). When the nematode was fed exclusively with *C*. *albicans*, we observed a rapid nuclear translocation of the transcription factor after two hours of incubation in the presence of the pathogen (Fig 6C). This translocation was maintained throughout the experiment, i.e., 76 h.

#### 3.4.3 Effect of Lcr35^®^ preventive treatment on DAF-16 nuclear translocation

**Fig 7:**
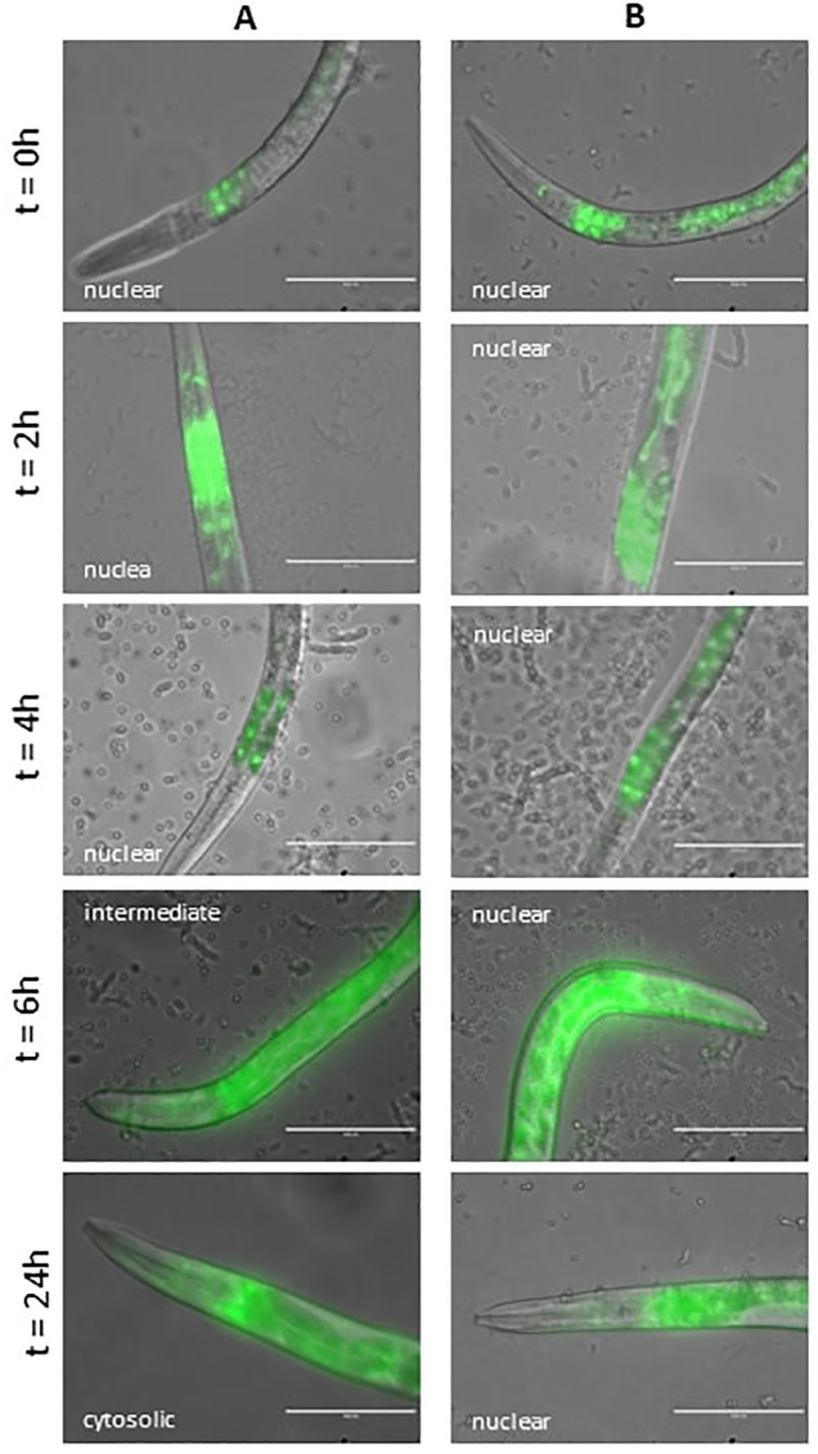
Impact of preventive Lcr35^®^ treatment on DAF-16 cellular localization in the *C*. *elegans* transgenic strain TJ-356 expressing DAF-16::GFP. Worms fed with *E*. *coli* OP50 + *C*. *albicans* (A) and on Lcr35^®^ + *C*. *albicans* (B). Scale bar, 100 μm.

We investigated the effect of preventive treatment on the cellular localization of DAF-16 over time after infection by *C*. *albicans* using the *C*. *elegans* DAF-16∷GFP mutant. When nematodes were first fed with *E*. *coli* OP50 before being infected, DAF-16 was fully observed in the nuclei up to 4 h after infection and then gradually translocated to the cytoplasm after 24 h (Fig 7A). Conversely, the worms that were first exposed to Lcr35^®^ and then to the pathogen showed a different response, and the transcription factor was found only in the nuclei (Fig 7B).

## 4 Discussion

The selection of microbial strains as probiotics is based on a combination of functional probiotic properties revealed first by classical basic *in vitro* testing. Beyond resistance to gastric pH or bile salts, the ability of a strain to adhere to epithelial cells is frequently studied since this represents a prerequisite for mucosal colonization as part of the anti-pathogen activity. Adhesion is also a key parameter for pathogens since it allows them to release toxins and enzymes directly into the target cell, facilitating their dissemination [49]. Nivoliez *et al*. showed that the native probiotic strain Lcr35^®^ adhered rather weakly to Caco-2 intestinal cells, while the industrial formulation increased this capacity [24]. We have demonstrated here the ability of Lcr35^®^ to inhibit the growth of the pathogen *C*. *albicans* and the formation of a *C*. *albicans* biofilm on an intestinal cell monolayer *in vitro*. As described by Jankowska *et al*., the low adherence of *L*. *rhamnosus* compared to *C*. *albicans* seems to reflect that competition for membrane receptors is not the only mechanism. It is probably related to the synthesis of antifungal effectors by the probiotic as well [49]. Exopolysaccharides (EPS) secreted by certain lactobacilli have been shown to modify the surface properties (hydrophobicity) of microorganisms with direct consequences on their adhesion capacities [50]. EPS have antifungal effects by inhibiting *C*. *albicans* growth and adhesion to epithelial cells. The surface polysaccharides of *L*. *rhamnosus* GG, a strain phylogenetically close to Lcr35, appear to interfere in the binding between the fungal lectin-like adhesins and host sugars or between the fungal cell wall carbohydrates and their epithelial adhesion receptor [51]. A recent study has shown that purified fractions of exopolysaccharides also interfered with adhesion capacities of microorganisms [52]. It would be interesting to assay the inhibitory properties of Lcr35^®^ EPS. However, to fully understand the probiotic mechanisms, *in vitro* approaches are too limited. Moving to an *in vivo* approach is mandatory to better understand the interactions between microorganisms (probiotics and pathogens) and the host response.

*C*. *elegans* is considered a powerful *in vivo* model for studying the pathogenicity of microorganisms [34,35,53–55] and the antimicrobial properties of lactic acid bacteria [56,57]. The nature of the nutrient source is an important parameter that has a great influence on nematode physiology. Regarding worm growth, it appears that there is some disparity depending on the type of lactic acid bacteria used to feed *C*. *elegans*. *Bifidobacterium spp*. had no influence on the size of adult worms, although their growth was slightly slowed down [58,59]. *Lactobacillus spp*. by contrast usually result in reduced growth rates and sizes and are sometimes even lethal to the larvae [60,61]. The mechanisms for explaining the longevity extension induced by lactic acid bacteria are not fully understood, but some authors have suggested the involvement of caloric restriction [62–64]. In our case, similar to the work of Komura *et al*., it seems that Lcr35^®^ did not induce pro-longevity effects through caloric restriction insofar as the growth of Lcr35^®^-fed nematodes is identical compared to *E*. *coli* OP50-fed worms [65].

After demonstrating the preventive effect of Lcr35 against *C*. *albicans* in the nematode, we decided to better understand the protective effect at the mechanistic level. In *C*. *elegans*, the insulin/IGF-1 signalling pathway is strongly involved in regulating the longevity and immunity of the animal. Signal transduction is mediated through DAF-16, a highly conserved FOXO transcription factor [66]. Using the GFP fusion protein, we have shown that Lcr35^®^ induces translocation of DAF-16 to the nucleus, suggesting that DAF-16 is involved in the probiotic mechanisms of action of Lcr35^®^. According to several studies, our data suggested that the pro-longevity effect of Lcr35^®^ implements mechanisms involving different regulatory pathways linked to DAF-16, such as the DAF-2/DAF-16 insulin pathway [67] or the c-Jun N-terminal kinase JNK-1/DAF-16 pathway [59]. The absence of modulation of *daf-2* expression in the presence of Lcr35^®^ suggests that the DAF-2/DAF-16 pathway is not involved and that the anti-*Candida* capacity of Lcr35^®^ is due to the JNK signalling pathway. The involvement of these pathways needs to be followed at proteomic and phosphoproteomic levels to validate this hypothesis.

The yeast *C*. *albicans* is capable of inducing a severe infection in *C*. *elegans*, causing a rapid death of the host and even after a very short contact time. This infection is first manifested by the colonization of the whole intestinal lumen by yeasts and then by the formation of hyphae piercing the cuticle of the nematode leading to its death [34,68]. In addition, it has been shown that strains of *C*. *albicans* incapable of forming hyphae, such as SPT20 mutants, have a significantly reduced pathogenicity in *C*. *elegans* as well as in *Galleria mellonella* or *Mus musculus* models while still being lethal [37]. In the nematode, it seems that the distention of the intestine caused by the accumulation of yeast is one of the causes of the death of the animal [35]. Recently, de Barros *et al*. [38] showed that *L*. *paracasei* 28.4 had anti-*C*. *albicans* activity both *in vitro* and *in vivo* by inhibiting filamentation of yeast protecting the nematode. Although *C*. *albicans* ATCC 10231 is able to form hyphae during *in vitro* assays, it failed to kill *C*. *elegans* by filamentation. Therefore, it is likely that Lcr35^®^ represses virulence factors in yeast other than filamentation.

From a mechanistic point of view, several hypotheses can explain the anti-*C*. *albicans* properties of Lcr35^®^ in the nematode: a direct interaction between the two microorganisms as well as an immunomodulation of the host by the probiotic. According to Nivoliez *et al*. demonstrating the inhibitory capacity of Lcr35^®^ with respect to the pathogen during a co-culture experiment [24], our data showed *Candida albicans* inhibition on mammalian cell monolayers. This inhibition may be due to nutrient competition (i.e., glycogen consumption) or to the production of toxic metabolites against the yeast [24]. We have shown that even after preventive treatment with the probiotic, the digestive tract of the nematode is colonized by the pathogen without showing a pathological state. This suggests that Lcr35^®^ induced repression of virulence factors in *C*. *albicans*, as shown by De Barros *et al*. [38]. Moreover, an *in vitro* study on human dendritic cells revealed that Lcr35^®^ induced a large dose-dependent modulation not only in the expression of genes mainly involved in the immune response but also in the expression of CD, HLA and TLR membrane proteins. Highly conserved and found in *C*. *elegans*, TLR also plays a role in the antipathogenic response of the nematode by activating the p38 MAPK pathway [59]. A pro-inflammatory effect has also been shown through cytokine secretion, such as IL-1β, IL-12, TNFα. However, this immunomodulation takes place only in the presence of a high concentration of Lcr35^®^ [69]. In *C*. *elegans*, DAF-16 is closely related to mammalian FOXO3a, a transcription factor involved the inflammatory process [70]. Therefore, nuclear translocation of DAF-16 by Lcr35^®^ can be interpreted as the establishment of an inflammatory response in the host allowing it to survive an infection. In our study, we observed that the duration of the Lcr35^®^ treatment influences the preventive anti-*C*. *albicans* effect on nematode lifespan, suggesting that the quantity of Lcr35^®^ ingested and/or the treatment period of time may have an impact on the efficiency of the treatment. A thorough transcriptional study will be interesting to characterize the dose-dependent effect probiotics administered. We demonstrated that Lcr35^®^ induces a transcriptional response in the host by activating the transcription factor DAF-16 and repressing the p38 MAPK signalling pathway, including in the presence of *C*. *albicans*. We also observed the repression of the genes encoding antimicrobials when fungal infection was preceded by probiotic treatment. The work of Pukkila-Worley *et al*. [35] demonstrated that *C*. *albicans* induced a fast antifungal response in the host inducing the expression of antimicrobial genes such as *abf-2*, *cnc-4*, *cnc-7*, *fipr-22* and *fipr-23*. With the exception of *abf-2*, all these genes are under the control of PMK-1, whose inactivation makes the nematode susceptible to infection. In our study, we showed that an Lcr35^®^ preventive treatment induced a down-regulation in the *cnc-4*, *fipr-22* and *fipr-23* genes, while *pmk-1* remained unchanged compared to the control condition. Based on the data of Pukkila-Worley *et al*., the absence of overexpression of these genes in the presence of *C*. *albicans* after pre-exposure with Lcr35^®^ suggests again that the probiotic inhibits yeast virulence, obviating the establishment of a defence mechanism by the host. Similar results have also been observed with *Salmonella* Enteritidis, where the authors hypothesize that the probiotics used induce immunotolerance in the nematode rather than the synthesis of antimicrobials [58]. The use of *C*. *elegans* mutants or RNAi could be further considered to decipher the signalling and regulation mechanisms.

## 5 Conclusion

This study demonstrates the preventive anti-*C*. *albicans* properties of Lcr35^®^ using both *in vitro* and *in vivo* models. The probiotic strain inhibits the growth of the pathogenic yeast and its ability to form biofilms on intestinal cells *in vitro*. Lcr35^®^ allows protection of the host *C*. *elegans* against infection despite the presence of *C*. *albicans* in its gut. Lcr35^®^ during *C*. *albicans* infection seems to induce a decrease in the immune response of the nematode (downregulation of *sek-1*, *pmk-1*, *abf-2*, *cnc-4* and *fipr-22*/*23*). Extra studies on *C*. *elegans* whole transcriptome modulation by Lcr35^®^ would be interesting to further reveal other mechanisms involved. The study of the yeast virulence gene modulation induced by Lcr35^®^ could be very informative about the complex mechanisms of the probiotic mechanisms of action. Additionally, in a second phase, the realization of a comparative study between Lcr35^®^ and other *Lactobacillus* strains (*L*. *rhamnosus*, *L*. *casei*, *L*. *paracasei*) as well as between different strains of *CC*. *albicans*, including clinical strains, could be of interest to determine the degree of strain dependence of our results.
